# Public Transcriptomic Data Meta-Analysis Demonstrates TAAR6 Expression in the Mental Disorder-Related Brain Areas in Human and Mouse Brain

**DOI:** 10.3390/biom12091259

**Published:** 2022-09-07

**Authors:** Anastasia N. Vaganova, Nataliia V. Katolikova, Ramilya Z. Murtazina, Savelii R. Kuvarzin, Raul R. Gainetdinov

**Affiliations:** 1 Institute of Translational Biomedicine, Saint Petersburg State University, Universitetskaya nab. 7/9, 199034 Saint Petersburg, Russia; 2 St. Petersburg University Hospital, Saint Petersburg State University, Universitetskaya nab. 7/9, 199034 Saint Petersburg, Russia

**Keywords:** trace amines, trace amine-associated receptor, TAAR, TAAR6, human brain, limbic brain, prefrontal cortex, nucleus accumbens, transcriptomic data

## Abstract

G protein-coupled trace amine-associated receptors (TAAR) recognize different classes of amine compounds, including trace amines or other exogenous and endogenous molecules. Yet, most members of the TAAR family (TAAR2-TAAR9) are considered olfactory receptors involved in sensing innate odors. In this study, TAAR6 mRNA expression was evaluated in the brain transcriptomic datasets available in the GEO, Allen Brain Atlas, and GTEx databases. Transcriptomic data analysis demonstrated ubiquitous weak TAAR6 mRNA expression in the brain, especially in the prefrontal cortex and nucleus accumbens. RNA sequencing of isolated cells from the nucleus accumbens showed that the expression of TAAR6 in some cell populations may be more pronounced than in whole-tissue samples. Curiously, in D1 and D2 dopamine receptor-expressing medium spiny GABAergic neurons of the nucleus accumbens, TAAR6 expression was co-regulated with genes involved in G protein-coupled receptor signaling. However, in cholinergic interneurons of the nucleus accumbens, TAAR6 expression was not associated with the activation of any specific biological process. Finally, TAAR6 expression in the mouse prefrontal cortex was validated experimentally by RT-PCR analysis. These data demonstrated that TAAR6 is expressed at low levels in the human and mouse brain, particularly in limbic structures involved in the pathogenesis of mental disorders, and thus might represent a new pharmacotherapeutic target.

## 1. Introduction

Along with classical monoamine neurotransmitters, like catecholamines or 5-hydroxytryptamine, some other biogenic amine compounds with a similar structure are found in the synaptosomal fraction of neurons. These molecules, such as beta-phenylethylamine, tyramine, tryptamine, and others, are present in low concentrations (1–100 ng. per g.), at levels significantly lower compared to the monoamine transmitters, and thus are named “trace amines” [1]. G protein-coupled trace amine-associated receptors (TAARs) recognize both trace amines and some other endogenous and exogenous amine molecules [2]. Humans have six functional members of the TAAR family (TAAR1, TAAR2, TAAR5, TAAR6, TAAR8, and TAAR9). Whereas TAAR1 expression and function in the brain have been firmly established, all other TAARs until recently were considered olfactory receptors sensing innate odors [2,3]. However, recent studies employing knockout mice and human transcriptomic data analyses revealed TAAR2 and TAAR5 expression in several brain areas where these receptors modulate emotional behaviors, sensorimotor functions, and adult neurogenesis [4,5,6,7].

A TAAR gene family is present in all jawed vertebrates [8]. However, the TAAR6 receptor gene appeared only after the divergence of placental Mammalia from the common ancestor with marsupials [9]. TAAR6 is suggested to have allosteric and orthosteric ligand-binding sites. Ligands that bind to allosteric sites act as modulators and could affect the interaction with the orthosteric ligand. However, until now, no allosteric modulators have been described for any TAAR [10].

In transfected cells, rhesus monkey TAAR6 did not alter intracellular cAMP levels, MAPK/ERK phosphorylation, or cell impedance in response to 10 μM of dopamine, norepinephrine, serotonin, beta-phenylethylamine, octopamine, tryptamine, tyramine, or methamphetamine, so these molecules likely are not TAAR6 agonists [11]. In silico modeling shows TAAR6 interaction with aliphatic ligands, like putrescine or cadaverine [10]. Likewise, another in silico study demonstrated that conservative aspartate or glutamate residues on the extracellular TAAR6 part facilitates the transition of cadaverine or putrescine from the extracellular aqueous environment to the binding site [12]. A recent study indicated that in vitro TAAR6 is not affected by cadaverine or putrescine but can be activated by tertiary amine N,N-dimethylcyclohexylamine at high micromolar concentrations, and these results are consistent with the hypothesis that TAAR6 is evolutionarily tuned toward tertiary amines [13,14]. Hence, it was suggested that TAAR6 might represent one of the endogenous hallucinogen receptors for N,N-dimethyltryptamine, 5-hydroxy N,N-dimethyltryptamine (bufotenine), or 5-methoxy-N,N-dimethyltryptamine [15]. Even so, no endogenous TAAR6-specific ligands have been found so far.

Interestingly, TAAR6 is considered a potential target of antipsychotic drugs. The analysis of aripiprazole treatment effectiveness indicated that some TAAR6 alleles in the genotype of schizophrenic patients correlate with improved response to aripiprazole therapy [16]. In the meantime, in silico comparative molecular docking approaches showed that aripiprazole and other antipsychotic drugs might bind to the extracellular TAAR6 region [17].

TAAR6 is well recognized as being expressed in the olfactory system. It is located on the dendrites and in perinuclear compartments of the olfactory sensory neurons, where it is co-localized with the endoplasmic reticulum and Golgi apparatus and in corresponding glomeruli of the olfactory bulbs [18,19,20,21]. At the same time, TAAR6 expression in the central nervous system remains controversial. Initial investigations have indicated its low expression in the brain regions implicated in the pathophysiology of schizophrenia, including the basal ganglia, frontal cortex, substantia nigra, amygdala, and hippocampus [22,23], but later studies do not support these findings. For example, no TAAR6 mRNA was found in the mouse brain [21] or monoaminergic brain areas in rhesus monkeys [11], leading to questions about its involvement in brain functions. Although the current views on TAAR6 expression in the brain remain controversial, several TAAR6 gene polymorphisms have been associated with mental diseases, including schizophrenia and bipolar affective disorder [24,25,26,27,28,29,30], which indirectly indicates the involvement of TAAR6 in brain functioning. Thus, the objective of the present study is a detailed evaluation of TAAR6 expression in the human and mouse brain transcriptomic data currently available in public databases. To estimate the level of TAAR6 expression, we compared it with values of mRNA transcription of other GPCRs with well-established expression profiles in the brain, such as the dopamine D2 receptor (DRD2), serotonin 5-HT1A receptor (HTR1A), and beta-2 adrenergic receptor (ADRB2). We also validated the TAAR6 mRNA expression data by reverse-transcription PCR (RT-PCR) analysis in the mouse brain.

## 2. Materials and Methods

### 2.1. Data Collection and Inclusion Criteria for Datasets

Transcriptomic data were retrieved from the National Center of Biotechnology Information (NCBI) Gene Expression Omnibus (GEO) database [31]. GEO Browser (available at https://www.ncbi.nlm.nih.gov/geo/browse/, accessed on 23 March 2022 [32]) was searched for the terms ”brain”, “cortex”, “cortical”, “hippocampus”, “hippocampal”, “striatum”, “striatal”, “caudatus”, “putamen”, “accumbens”, “raphe”, “brainstem”, “pons”, “cerebellum”, “cerebellar”, “basal ganglia”, and “white matter” through 15 February 2022. Each GEO dataset included in the analysis meets the following criteria: (1) human or mouse RNAseq study; (2) TAAR6 values are available, expression data are represented in raw counts or counts per million (CPM); (3) clear explanation of sample origin (i.e., data were excluded if the part of the brain or cortex was not specified); (4) at least 5 samples from healthy adult subjects/non-treated control animals per dataset; (5) at least 40 million reads per SRA file in healthy subject/control animal groups and minimal average number of reads in the SRA files per dataset is not less than 47.5 million reads. Studies based on the targeted purification of the polysomal mRNA (TRAP-Seq) or datasets, which contain only embryo/fetal samples, were excluded. After exclusion of non-relevant datasets, 5 human datasets and 12 mouse datasets were selected for the analysis (Table 1, Table 2, Tables S1 and S2).

Additionally, we included in the review two mouse datasets and one human transcriptomic dataset, which consist of data on the RNA isolated from the fractionated neurons or subcellular structures (Table 3 and Table S3). The GSE130376 dataset comprises the data for nine single cholinergic neurons isolated from the nucleus accumbens. The neurons were isolated from mice that are susceptible or resilient hereditary to cocaine addiction. Only three animals were wild type, so the dataset did not fully meet the inclusion criteria for the number of samples. Another dataset, GSE121199, comprises transcriptomic data for D1 or D2 dopamine receptor-positive medium spiny neurons (MSNs) of the mouse nucleus accumbens (D1-MSN and D2-MSN, respectively). The samples in this dataset were sequenced with a depth lower than what we selected to meet the inclusion criteria. However, the RNA-sequencing results of these studies are quite informative to reveal TAAR6 expression, probably because of the experimental design involving cell fractionation. The human dataset GSE110727, which includes cytoplasmic and nuclear RNA isolated from the human frontal cortex samples, also was reviewed in this part of the meta-analysis.

### 2.2. Raw Data Normalization and Analysis

For uniform estimation of the expression levels, all data were CPM-normalized by the cpm function in the edgeR package [33]. CPM values above the threshold level equal to 10/L, where L is the library size in millions, were considered positive. The distribution of CPM-normalized expression levels was analyzed and visualized by the beeswarm R package.

### 2.3. Gene Ontology Terms Enrichment Analysis of TAAR6 Co-Genes

TAAR6 co-expressed gene clusters were identified by the Pearson correlation coefficient (*p* < 0.05). The top 500 co-expressed genes were selected for the gene ontology (GO) enrichment analysis. GO enrichment analysis (identification of GO terms that are significantly enriched by the genes of the selected set) was performed in the identified co-expressed gene clusters and visualization of results was performed by the ShinyGo 0.76 [34] web tool (available at http://bioinformatics.sdstate.edu/go/, accessed on 29 June 2022). The “GO Biological Process” (BP) and “GO Molecular Function” (MF) databases were applied. GO terms with a false-discovery rate (FDR) <0.005 were considered significantly enriched.

### 2.4. Genotype-Tissue Expression (GTEx) Data

GTEx transcriptomic data were obtained from dbGaP accession number phs000424.v8.p2 on 22 March 2022. Data were visualized by the GTEx Portal interactive interface available at https://www.gtexportal.org/home/ (accessed on 22 March 2022) [35].

### 2.5. Public In Situ Hybridization (ISH) Data

The ISH data were received from the Allen Brain Map (available at https://portal.brain-map.org, accessed on 16 March 2022 [36]). TAAR6 expression analysis in mouse brain was conducted using data obtained from the Allen Mouse Brain Atlas (https://mouse.brain-map.org, accessed on 16 March 2022 [37,38,39,40,41]). As ISH data for the human brain in the Allen Human Brain Atlas (https://human.brain-map.org/, accessed on 23 March 2022 [42]) did not contain the TAAR6 data for the whole brain; we did not include it in this study.

TAAR6 expression data were collected from the experiment 70724989, riboprobe RP_050705_03_A11, sagittal projection (available at https://mouse.brain-map.org/experiment/show/70724989, accessed on 16 March 2022 [43]). Adrb2 expression was estimated in the experiment 68744522, riboprobe RP_050208_01_A11, sagittal projection (available at https://mouse.brain-map.org/experiment/show/68744522, accessed on 16 March 2022 [44]). Drd2 expression was estimated in experiment 358, riboprobe RP_Baylor_102735, sagittal projection (available at https://mouse.brain-map.org/experiment/show/358, accessed on 16 March 2022 [45]). Htr1a expression was estimated in the experiment 79394355, riboprobe RP_071018_03_D06, sagittal projection (available at https://mouse.brain-map.org/experiment/show/79394355, accessed on 16 March 2022 [46]).

Based on “Technical white paper: in situ hybridization data production” and “Technical white paper: informatics data processing” from the Allen Institute of Brain Science, colorimetric data were obtained from scans made by the Tecan robot with GenePaint technology. Every Tecan-automated ISH run contains a positive control section and a negative control section. Quality control for each ISH-stained section image includes the examination of focus, lighting, artifacts (including bubbles and debris), overall tissue quality, and an acceptable standard for consistency, including anatomic normalcy, dissection quality, section orientation, and signal-to-noise ratio. In some cases, the consistency with published literature or other evidence for expression was also examined. The global adaptive thresholding method was used in the resulting slides for the separation of the background and foreground.

Raw expression data were used to assess the expression level of genes of interest in the mouse brain.

### 2.6. Tissue Sampling and RNA Extraction

Experiments were performed in 3–4-month-old male C57BL/6N mice. The animals (*n* = 6) were euthanized using cervical dislocation. The prefrontal cortex, hippocampus, olfactory bulb, striatum, hypothalamus, cerebellum, and brainstem were dissected on ice. To extract the main olfactory epithelium, the skin was removed and the head was split along the sagittal plane into left and right hemispheres. Both the dorsal and the ventral-sight orange/brown epithelia were carefully removed from the upper posterior one-third of the nasal cavity. The main olfactory epithelium was sampled as a positive control for TAAR6 expression. Tissues were immediately frozen in liquid nitrogen and stored at −80 °C.

RNA was isolated from tissues with TRI Reagent (MRC) according to the manufacturer’s protocol, dissolved in RNase-free water, and kept at −80 °C until use. RNA concentration was quantified using spectrophotometry (NanoDrop Technologies, Wilmington DE, USA). To eliminate any remaining genomic DNA, the TURBO DNA-free kit (Thermo Fisher Scientific, Waltham, MA USA) was used on RNA samples. As a control for the successful removal of genomic DNA, each sample was exposed to the same treatment, except that the reverse transcriptase was not added (RT^-^control).

### 2.7. RT-PCR Analysis

1 μg of RNA was taken for the synthesis of cDNA using Revertaid Reverse Transcriptase (Thermo Fisher Scientific, Waltham, MA, USA). Briefly, 1 U of reverse transcriptase, reaction mix containing 3 μM random hexamer primers (Beagle, Russia), and 1 μL of RiboLock RNase Inhibitor (Thermo Fisher Scientific, Waltham, MA, USA) were added to DNase-treated RNA and exposed to the following protocol: annealing at 25 °C for 10 min, transcription at 42 °C for 90 min, and termination at 70 °C for 10 min. The volume of reverse transcription reaction was 30 μL. The cDNA samples were stored at −80 °C. One µL of cDNA was used for PCR. Reactions were performed in duplicate using qPCRmix-HS SYBR (Evrogen, Russia) under the following condition: 95 °C for 5 min followed by 35 cycles of 95 °C for 20 s, 60 °C for 20 s, and 72 °C for 30 s. PCR was conducted in a CFX96 instrument using CFX Manager software (Bio-Rad, Hercules, CA USA).

Primers to detect mouse TAAR6 were as follows: mTAAR6F 5′-tctctcttccacctgagctt-3′, mTAAR6R 5′-gacacagacaccgtgaactt-3′, and product length 92 b.p. [47]. The primers were checked for specificity by PCR with mouse genome DNA, plasmid containing mouse Taar6 gene, and cDNA of the mouse olfactory epithelium, which constitutively express TAAR6 [47]. The amplification specificity was confirmed by melting-curve analysis (from 55 to 95 °C) and 2% agarose gel electrophoresis in sodium borate buffer. A single peak on the melting curve was observed in all controls and samples. The Gel Doc XR+ Documentation System (Bio-Rad) was used for agarose EtBr-stained gel analysis. The mouse glyceraldehyde 3-phosphate dehydrogenase (GAPDH) gene (primers: mGAPDH_F2: 5′-ttgatggcaacaatctccac-3′, mGAPDH_R2: 5′-cgtcccgtagacaaaatggt-3′), which is a housekeeping gene, was included as a quality control for RNA isolation and cDNA synthesis.

All procedures involving animals and their care were carried out following the guidelines established by the European Community Council (Directive 2010/63/EU on 22 September 2010) and were approved by the Ethics Committee of St. Petersburg State University, St. Petersburg, Russia.

## 3. Results

### 3.1. TAAR6 Expression in the Brain Neocortical Areas and Hippocampus in Datasets from GEO

**Figure 1:**
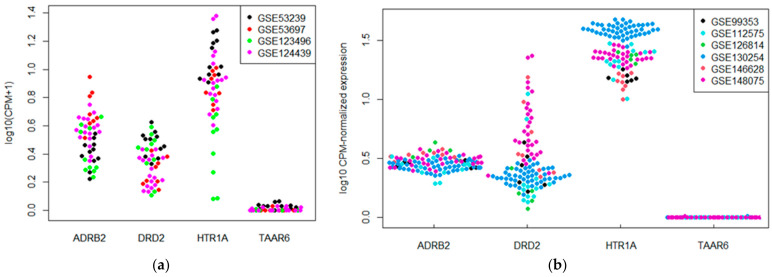
Expression levels of TAAR6, DRD2, ADRB2, and HTR1A in the neocortical tissues in (**a**) human and (**b**) mouse.

Four whole-tissue human transcriptomic datasets and 10 whole-tissue mouse transcriptomic datasets, which were included in the meta-analysis, represented the data for the TAAR6 expression patterns in the different cortical areas—in particular, in the prefrontal cortex, motor cortex, frontal cortex, parietal cortex, and hippocampus. TAAR6 expression data were available in the human neocortical structures, i.e., the prefrontal cortex (*n* = 19), motor cortex (*n* = 8), frontal cortex (*n* = 14), and parietal cortex (*n* = 5). All datasets included at least one sample per set that expressed TAAR6. In the GSE53239 dataset, which represented expression profiles in the dorsal prefrontal cortex, TAAR6 expression was the most pronounced. The expression levels of TAAR6 above the cut-off were present in three of 11 samples (27%). The same trend was detected in the other dataset, GSE53697, where only one of eight dorsal prefrontal cortex samples demonstrated TAAR6 expression above the cut-off level (Figure 1a). This discrepancy is possibly the consequence of a significant difference in the sequencing depth between these datasets. Whereas most datasets had a mean sequence depth of <100 million reads per sample, only the GSE53239 dataset had a mean sequencing depth of >150 million reads per sample. Therefore, TAAR6 expression levels in the frontal, motor, and parietal cortical areas were below cut-off, possibly because of the insufficient sequencing depth.

The expression of TAAR6 in mouse neocortical structures seemed to be even less detectable. Six datasets that showed transcriptional profiles in the prefrontal cortex (*n* = 89) and frontal cortex (*n* = 19) were included in the review. Most studied samples were TAAR6-negative, and only two prefrontal cortex samples expressed TAAR6 at the levels above the cut-off (Figure 1b).

Data for TAAR6 expression in the hippocampus were available for only five human samples in the single GSE123496 dataset. Five mouse transcriptomic datasets provided data on TAAR6 expression in the hippocampal samples (*n* = 61). No TAAR6 expression was found in the mouse samples. In the human specimens, TAAR6 was expressed in four of five samples, but the expression levels did not reach cut-off levels in any of them (data not shown).

Next, to make a quantitative assessment of the TAAR6 mRNA transcription level, we additionally evaluated the mRNA content of three other GPCRs that are well known to be expressed in the brain, including DRD2, HTR1A, and ADRB2. In the same datasets, DRD2, HTR1A, and ADRB2 were expressed at low levels (i.e., 0.5–10 CPM) in most neocortex (Figure 1a,b) and hippocampus samples (data not shown).

### 3.2. TAAR6 Expression in the Basal Ganglia in Datasets from GEO

**Figure 2:**
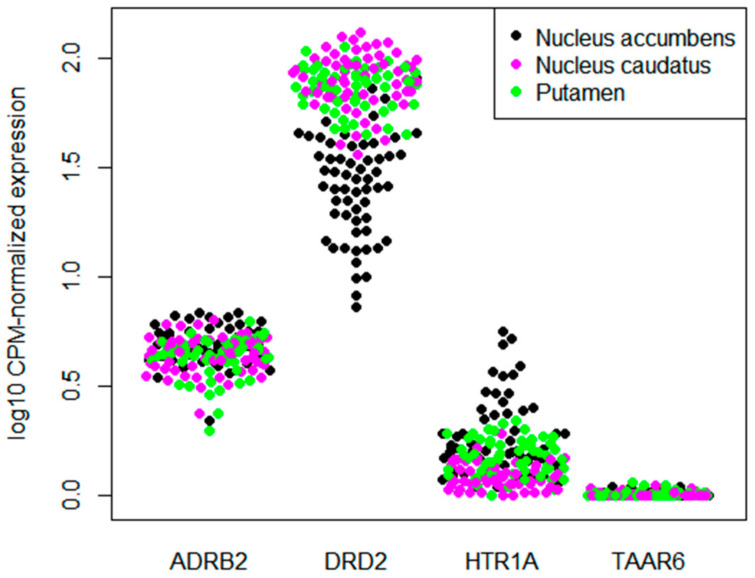
Expression levels of TAAR6, DRD2, ADRB2, and HTR1A in the human basal ganglia (dataset GSE160521).

One human transcriptomic dataset and four mouse datasets represented in the GEO database contained the data for TAAR6 expression in the basal ganglia. In the human dataset GSE160521, which summarized transcriptomic data for the nucleus accumbens (*n* = 59), caudate nucleus (*n* = 59), and putamen (*n* = 59), only an occasional TAAR6 expression below cut-off values was revealed (Figure 2). In addition, no TAAR6 expression was detected in the mouse datasets, including whole striatum samples (*n* = 23) and nucleus accumbens samples (*n* = 48). Only one of the studied nucleus accumbens samples was positive for TAAR6 expression at a level above the cut-off. As in the cortical samples, DRD2, ADRB2, and HTR1A were expressed in the basal ganglia at the low (i.e., 0.5–10 CPM) or medium (10–28 CPM) levels.

### 3.3. TAAR6 mRNA Distribution in the Brain Neuronal or Subcellular Fractions

Indeed, the data acquired by the separated cells or cell fractions RNA-seq appeared to be more informative than the whole-tissue data for TAAR6 expression in the brain regions. Three GEO datasets illustrated mRNA expression in the different neuronal fractions in the nucleus accumbens, or in cytoplasmic and nuclear fractions in the frontal cortex.

**Figure 3:**
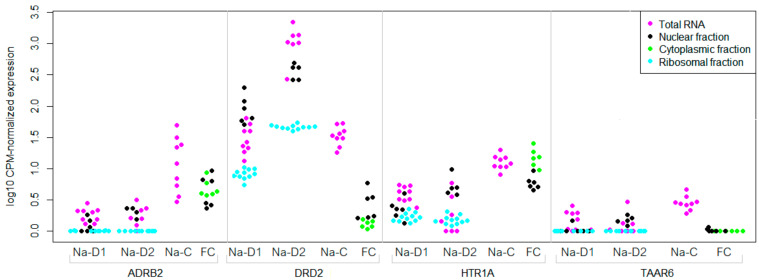
Expression levels of TAAR6, DRD2, ADRB2, and HTR1A in the subcellular structures in the mouse nucleus accumbens (GSE121199 and GSE130376) and human frontal cortex (GSE110727). Na-D1—expression in the D1 dopamine receptor positive medium spiny neurons of the nucleus accumbens, Na-D2—expression in the D2 dopamine receptor positive medium spiny neurons of the nucleus accumbens, Na-C—expression in the cholinergic neurons of the nucleus accumbens, FC—expression in the subcellular structures of the human frontal cortex.

In particular, TAAR6 was detected in all studied cholinergic neurons of the nucleus accumbens in the GSE130376 mouse dataset. In the GSE121199 dataset, total RNA from the mouse nucleus accumbens MSNs was compared with nuclear RNA or RNA, which was bound with ribosomes in these cells. TAAR6 was expressed (i.e., had a CPM value above cut-off) in D1-MSNs (5 of 8 whole-cell samples and 1 of 5 nuclear samples) and D2-MSNs (2 of 7 whole-cell samples and 4 of 5 nuclear samples). All neuronal fractions expressed ADRB2, DRD2, and HTR1A mRNA receptors, and in both groups of MSNs, DRD2 expression was higher than in other receptors (Figure 3). In contrast, TAAR6 mRNA was detected only in total cellular RNA samples or in the nuclear mRNA. No TAAR6 mRNA was detected in ribosome-associated RNA samples isolated from D1- or D2-MSNs.

A weaker but congruent TAAR6 expression pattern in the dataset GSE110727 comprised transcriptomic data for cytoplasmic and nuclear RNA in the human frontal cortex. Only two samples with low TAAR6 expression were identified in this dataset (and only one of them demonstrated an expression level above the cut-off), and in both cases, TAAR6 expression was revealed in the nuclear transcriptome. In addition, as was described previously, DRD2 mRNA was predominately associated with the nucleus and HTR1A mRNA was mostly detected in the cytoplasm [48,49] (Figure 3).

### 3.4. Functional Analysis of Genes Co-Expressed with TAAR6 in Different Neuronal Populations in the Mouse Nucleus Accumbens Samples

To estimate the functional significance of TAAR6 expression in different cell populations of the mouse nucleus accumbens, we selected and analyzed the TAAR6 ещз 500 co-expressed genes in different cell groups (i.e., in the whole-cell D1- or the nuclear fraction of D2-MSNs described in the dataset GSE121199, and the cholinergic interneurons described in GSE130376; Table S4). All genes selected for the analysis were significantly co-expressed with TAAR6 (*r* > 0.85, *p* < 0.05).

**Figure 4:**
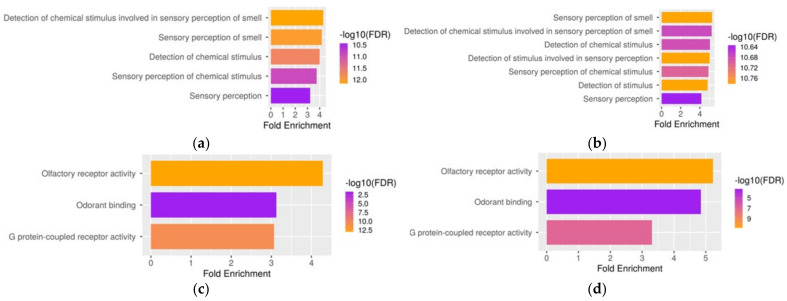
GO terms enriched by genes co-expressed with TAAR6 in the nucleus accumbens medium spiny neurons (GSE121199). (**a**) Biological-process GO terms enriched in top-500 TAAR6 co-expressed genes in the D1-medium spiny neurons; (**b**) biological-process GO terms enriched in top-500 TAAR6 co-expressed genes in the D2-medium spiny neurons; (**c**) molecular-function GO terms enriched in top-500 TAAR6 co-expressed genes in the D1-medium spiny neurons; (**d**) molecular-function GO terms enriched in top-500 TAAR6 co-expressed genes in the D2-medium spiny neurons. Bar colors indicate the false-discovery-rate (FDR) value. There was no significant GO term enrichment for TAAR6 co-expressed genes in cholinergic interneurons (GSE130376).

The results of GO enrichment in the TAAR6 co-expressed genes in D1- or D2-MSNs were congruent and demonstrated only slight differences. The BP terms describing the smell or chemical stimulus recognition were enriched in both gene sets. Likewise, MF terms enriched in TAAR6 co-expressed gene sets in D1- or D2-MSNs were similar. Regardless of the predominantly expressed dopamine receptor, in the MSNs, TAAR6 co-expressed genes were enriched with genes involved in the recognition of smell and other chemical stimuli (Figure 4a–d). At the same time, in cholinergic interneurons, the GO enrichment analysis did not reveal TAAR6 co-expression with any BP or MF terms (FDR > 0.05).

### 3.5. Genotype-Tissue Expression (GTEx) Data

**Figure 5:**
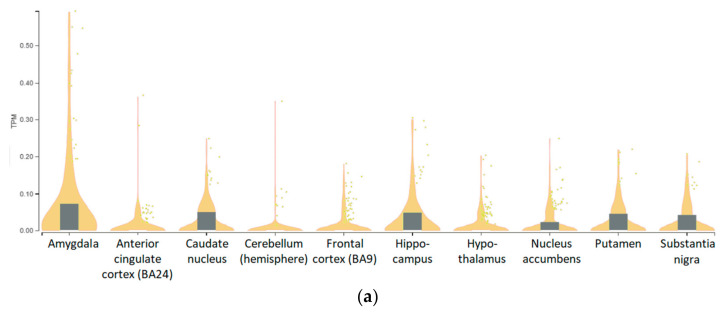

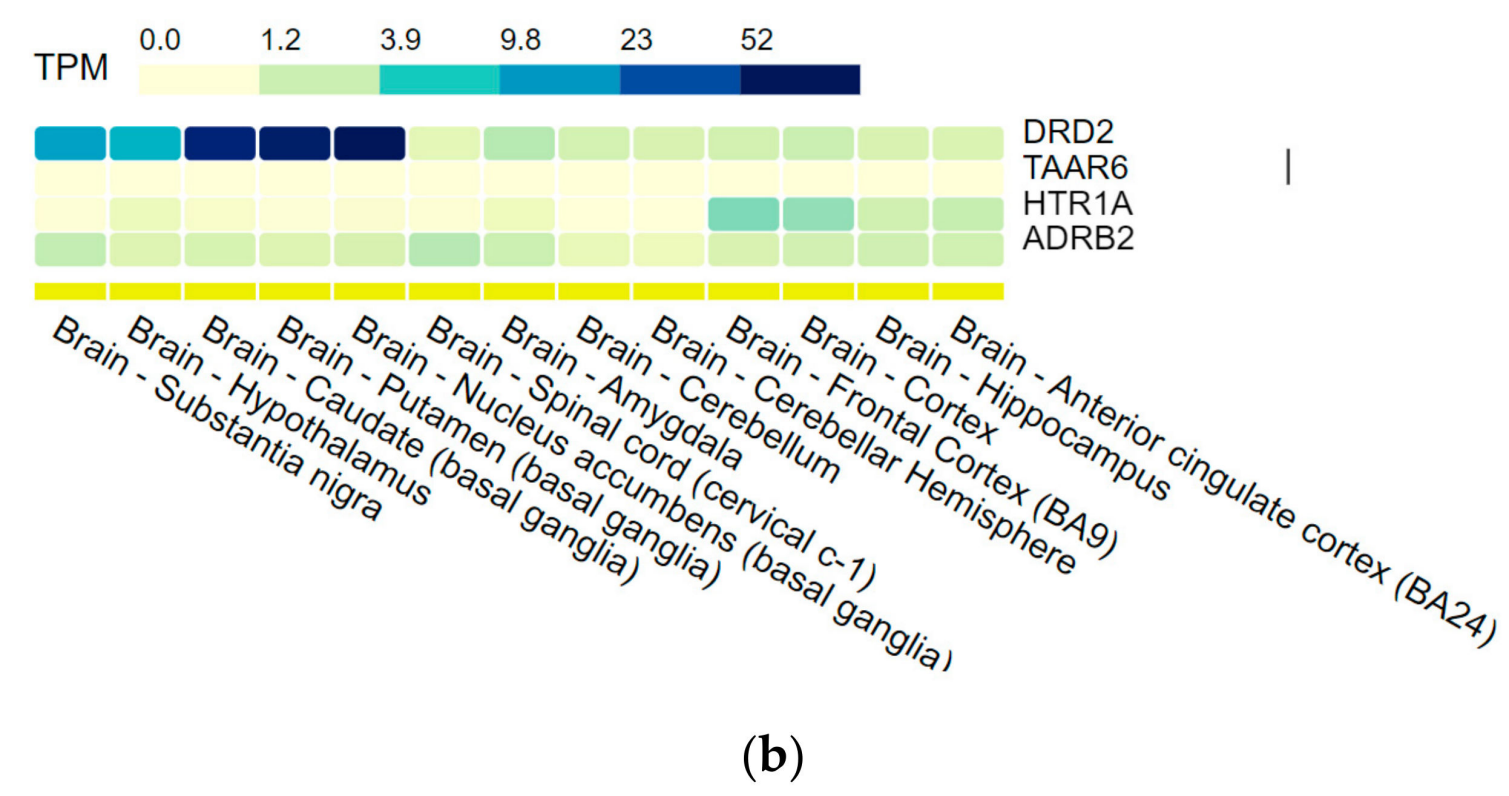
TAAR6 expression in the human samples from the GTEx database. (**a**) Violin boxplot for distribution of TAAR6 expression in the human brain regions in the GTEx Analysis V8 (dbGaP Accession phs000424.v8.p2) dataset, yellow violins depict distributions of numeric data of TAAR6 expression, the white line in the gray box plot shows the median value of the expression (commonly near the zero), yellow dots indicate the TAAR6 expression level in each sample; (**b**) matching of TAAR6 expression with GPCRs DRD2, ADRB2, and HTR1A. The pictures were generated by the GTEx Portal interactive interface.

The GTEx Analysis V8 (dbGaP Accession phs000424.v8.p2) dataset demonstrated TAAR6 expression in all studied human brain regions, including the amygdala, anterior cingulate cortex area BA24, caudate nucleus, cerebellar hemispheres, frontal cortex BA9 area, hippocampus, hypothalamus, nucleus accumbens, putamen, spinal cord (cervical c-1 level), and substantia nigra. Applying the cut-off level of 0.1 for transcripts-per-million (TPM) values, only accidental tissue samples may be considered positive in this dataset (Figure 5a). In sum, in the amygdala, the TAAR6 expression levels were the highest of the dataset, followed by the hippocampus, putamen, and nucleus caudatus. As in the previously described transcriptomic data, in the GTEx dataset, DRD2, ADRB2, and HTR1A were commonly higher expressed than TAAR6 (Figure 5b).

Although the GTEx data for cortex and basal ganglia are accordant with the data mined in the GEO database (Figure 5b), there were no relevant datasets to estimate TAAR6 expression in other regions. Thus, no TAAR6 expression was revealed in the selected datasets (Table 1 and Table 2) for samples from the cerebellum, hypothalamus, substantia nigra, ventral tegmental area, or ventral midbrain in GEO datasets included in the review.

### 3.6. ISH Visualization of TAAR6 Expression in the Mouse Brain

The ISH expression data were mined from the Allen Brain Atlas: Mouse Brain as described in the Material and Methods section. Following the represented raw expression values and section images, TAAR6 was weakly expressed in all estimated brain regions. 

**Figure 6:**
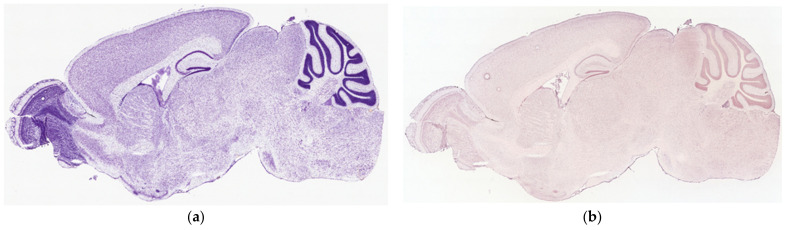

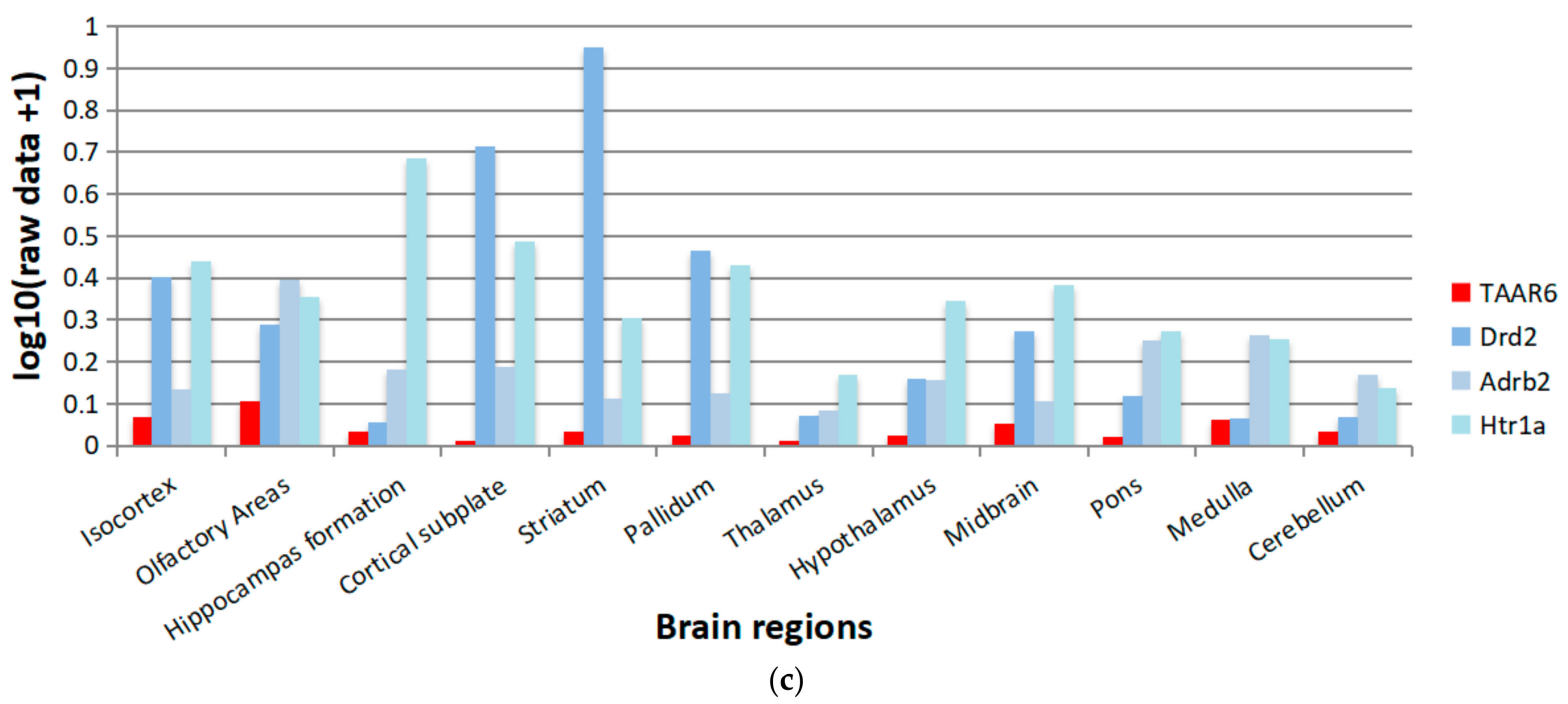
TAAR6 mRNA expression patterns in mouse brain, sagittal projection. (**a**) Nissl staining from the Allen Mouse Brain Atlas; (**b**) expression of the Taar6 gene in adult mouse brain identified by in situ hybridization in the Allen Mouse Brain Atlas (**b** and **c** in the same slice position); (**c**) expression of Taar6, Drd2, Adrb2, and Htr1a genes in the mouse brain, based on log10 data of expression from the Allen Mouse Brain Atlas. Data available at the Allen Mouse Brain Atlas, mouse.brain-map.org and atlas.brain-map.org. Nissl staining and TAAR6 riboprobe RP_050705_03_A11: https://mouse.brain-map.org/experiment/show/70724989 accessed on d16 March 2022; Adrb2 riboprobe RP_050208_01_A11, sagittal projection: https://mouse.brain-map.org/experiment/show/68744522 accessed on 16 March 2022; Drd2 riboprobe RP_Baylor_102735, sagittal projection: https://mouse.brain-map.org/experiment/show/358 accessed on 16 March 2022; Htr1a riboprobe RP_071018_03_D06, sagittal projection: https://mouse.brain-map.org/experiment/show/79394355 accessed on 16 March 2022.

The highest expression was shown in the murine olfactory bulb, which was not described in any other dataset included in the review. As in the RNAseq data, DRD2, ADRB2, and HTR1A expression levels were more pronounced than TAAR6 expression in all described structures (Figure 6).

### 3.7. TAAR6 mRNA Expression in the Mouse Brain

**Figure 7:**
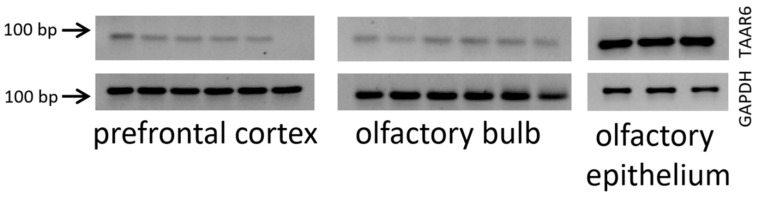
Reverse transcription-polymerase chain reaction (RT-PCR) with TAAR6- and GAPDH (housekeeping gene)-specific primers using RNA isolated from the mouse main olfactory epithelium, olfactory bulb, and prefrontal cortex confirmed TAAR6 expression in the prefrontal cortex. These gel images are cropped, and full-length images are presented in Supplementary Figure S1.

Finally, to validate public transcriptome data experimentally, we used RT-PCR measurements to analyze the expression of TAAR6 mRNA in the mouse prefrontal cortex, as the expression of TAAR6 was found in the most studied datasets, and compared it to expression in the olfactory system. We showed that TAAR6 mRNA is expressed in the prefrontal cortex and olfactory bulb as well as in the olfactory epithelium (Figure 7; Supplementary Figure S1). Specificity of TAAR6 RT-PCR analysis was confirmed using cDNA derived from the mouse olfactory epithelium, the site with the highest TAAR6 expression [21].

## 4. Discussion

The biological significance of TAAR6 in the brain remains uncertain. Nevertheless, it has attracted interest in psychiatric genetics. It was shown that the human TAAR6 gene is characterized by high variability. Thus, some researchers suggested that TAAR6 mutations may mirror its pseudogenization [23]. Although several studies have highlighted TAAR6 SNPs’ association with the risk of mental disorders [2,50], in particular with schizophrenia and bipolar affective disorder [23,25,26,27,28,29,30,51], it should be mentioned that the co-occurrence of TAAR6 gene polymorphisms with mental disorders remains controversial and several other studies failed to confirm such associations [52,53,54,55,56,57].

Low TAAR6 mRNA expression in humans was reported in the hippocampus, frontal cortex, substantia nigra, amygdala, and basal ganglia, but not in the cerebellum [23,30]; however, follow-up studies questioned the TAAR6 expression in the mammalian brain [11,23]. The present analysis of public transcriptomic data uncovered that TAAR6 mRNA is expressed in all examined brain regions but at low levels. The expression pattern of TAAR6 seems to be sporadic, which may be because of the limited number of cells expressing this receptor. TAAR6 expression in the prefrontal cortex and the nucleus accumbens seems to be the most proven, as it was revealed in the various GEO datasets, GTEx, and Allen Brain Atlas data. In the mouse prefrontal cortex, TAAR6 expression also was confirmed by RT-PCR. Analysis of TAAR6 expression in isolated neurons of the nucleus accumbens confirmed that the signal from specific cells in the tissue may be masked in the whole-tissue sample. Data from GTEx and the Allen Brain Atlas database also proved TAAR6 expression in the hippocampus, hypothalamus, cerebellum, amygdala, substantia nigra, olfactory bulb, thalamus, pons, whole midbrain, and medulla.

As the levels of TAAR6 mRNA expression in the nucleus accumbens neuronal populations are higher than in whole-tissue brain samples, we performed additional analysis of TAAR6 co-expressed genes in the brain region to explain the functional significance of TAAR6 expression. In the nucleus accumbens, GABAergic MSNs are divided into two types according to dopamine receptor expression (D1 or D2), and are the primary output neurons and make up 90–95% of all neurons in this structure [58]. Both MSN subpopulations modulate social behavior [59], stress-coping response [60], reward, and aversion [61]. It is known that the transcriptional profiles of D1- and D2-MSNs are largely similar [62]. Therefore, the similar functional significance of TAAR6-co-expressed genes in these neuronal populations is in line with the published data. TAAR6 receptor mRNA in MSNs is co-expressed with olfactory receptors (ORs). ORs are considered odorant receptors on olfactory sensory neurons, but there is some evidence for OR expression in different brain areas, including [61] the hippocampus, cerebellum, striatum, cortex, and medulla oblongata [63]. In isolated primary mesencephalic dopaminergic neurons, Olfr287 mediates the response to carvone and menthone [64]. Till now, the functional significance of ORs in MSNs had not been estimated. Both D1- and D2-MSN populations are heterogeneous. For example, MSNs vary in excitability in response to the stimulation [61]. It is possible that TAAR6 expression in MSNs is specific to any unidentified group of these cells or some of their functional states.

Cholinergic interneurons account for only 1–2% of the neurons in the nucleus accumbens and regulate MSN activity [58]. Monoamine signaling is essential for the functioning of these cells; in particular, abnormal DRD2 expression in these cells is associated with addiction [59]. No TAAR6 co-expression with DRD2 or statistically significant co-regulation with any functional gene group was revealed in this study, so the TAAR6 expression seems to be independent of any biological process in these cells. The obtained results may reflect the constitutive pattern of TAAR6 expression in the cholinergic interneurons. In addition, losing enrichment for any GO term may be the consequence of the biological heterogeneity of the study group, which comprised mice susceptible or resilient to cocaine addiction. Further analysis of the relation between TAAR6 expression levels and the functioning of different neurons in the nucleus accumbens may shed light on the pathogenesis of psychiatric disorders, as there are multiple lines of evidence of the involvement of the nucleus accumbens in these diseases [58,65].

Thus, expression of TAAR6 in the brain areas involved in mental disorders, such as the prefrontal cortex or nucleus accumbens, was found in this study. A low and predominantly limbic pattern of expression of TAAR6 is reminiscent of that of other TAAR receptors, such as TAAR1, TAAR2, and TAAR5 [4,5,6,7,49]. A recent analysis of human transcriptome data revealed that all TAARs are expressed in the limbic brain areas, with the highest expression level found for TAAR5 [49,66]. In a similar public data study [49], we also found that the level of TAAR5 expression was higher than that observed for TAAR6 in the present study. It should also be mentioned that despite the similarly low level of expression of TAAR1, the agonist of this receptor, Ulotaront, showed clinical efficacy in patients with schizophrenia, ameliorating both positive and negative symptoms without causing side effects of currently used antipsychotics [67,68,69,70].

Transcription is not the only factor influencing gene expression at the protein level. The resulting level of gene expression depends on multiple factors, including the RNA stability, modifications, and localization in the cell; the translation rate; and protein turnover, especially in the neurons, where the key aspects of the numerous gene expression are controlled locally in the cytoplasmic microdomains [71]. However, the obtained results demonstrate slightly higher levels of TAAR6 mRNA in the neuronal nuclei compared with the ribosomal or cytoplasmic fractions, which would indicate the possible significance of post-transcriptional regulation mechanisms like nuclear retention [72]. In neurons, TAAR6 is expressed on the cell surface [18], so the nuclear transcript localization and losing mRNA association with ribosomes could mirror the strict control of the sparse TAAR6 transcript translation to the protein. However, it was previously demonstrated that there is only a weak positive correlation between mRNA and protein levels in human tissues and that protein-to-mRNA levels vary significantly between the genes [73]. Therefore, it is not excluded that low-level TAAR6 mRNA gives rise to a significant amount of TAAR6 receptor because of the low turnover of the protein. Further assessment of post-transcription regulation of TAAR6 expression along with the research of this receptor abundance and turnover in the neurons and glial cells is necessary to determine whether low TAAR6 mRNA levels indeed reflect the low expression of this receptor.

There are several limitations in this study that must be acknowledged. The transcriptomic datasets generated with suitable sequencing depth for the number of brain regions are also unavailable in the GEO database, and TAAR6 expression data for these areas are accessible only in GTEx or the Allen Mouse Brain Atlas. This limits options for comparing the data independently reported by different research groups. Allen Mouse Brain Atlas [34] data are represented by ISH, which is a semi-quantitative method. The transcriptomic datasets included in the study were collected in different laboratories and cannot be completely standardized. Despite the measures to normalize the data, as was discussed in the Materials and Methods section, the full uniformity of the data and overcoming the batch effects are unattainable and may be a source of bias in this study. This limitation makes the complete differential expression analysis between datasets impossible, so the quantitative comparison of TAAR6 mRNA between the brain parts remain out of the scope of this meta-analysis.

## 5. Conclusions

The analysis of publicly available transcriptomic data demonstrates TAAR6 expression in several human brain regions, with predominant localization in the limbic brain area. In most brain regions, its expression is low and seems sporadic. Despite a low level of expression of this gene and a lack of data with the proper sequencing depth, it can also be found in mouse brain regions. TAAR6 mRNA presence in the mouse prefrontal cortex was also confirmed experimentally. Data generated by the sequencing RNA from separated cells or cell populations show that TAAR6 expression in some cells may be higher than the cut-off value but become masked in the whole-tissue sample. It should be noted that, depending on the group of neurons containing TAAR6 mRNA, its transcription may or may not be co-regulated with other genes. Thus, the pattern of TAAR6 expression in the brain, particularly in the areas involved in the pathogenesis of psychiatric disorders such as the frontal cortex and nucleus accumbens, supports previous genetic association studies and suggests that TAAR6 should be evaluated as a potential drug target to treat psychiatric disorders.

## Figures and Tables

**Table 1:** Human transcriptomic GEO datasets meeting the inclusion criteria and included in the analysis.

Accession Number	Title	Samples	*n* ^1^
GSE53239	RNA-sequencing of the brain transcriptome implicates dysregulation of neuroplasticity, circadian rhythms, and GTPase binding in bipolar disorder	Dorsolateral prefrontal cortex	11
GSE53697	RNAseq in Alzheimer’s disease patients	Dorsolateral prefrontal cortex	8
GSE123496	Human brain tissues from healthy controls and multiple sclerosis patients	Frontal cortex	5
		Parietal cortex	5
		Hippocampus	5
		Corpus callosum	5
		Internal capsule	5
GSE124439	Postmortem Cortex Samples Identify Distinct Molecular Subtypes of ALS: Retrotransposon Activation, Oxidative Stress, and Activated Glia	Frontal cortex	9
		Motor cortex	8
GSE160521	Diurnal rhythms across the human dorsal and ventral striatum	Nucleus accumbens	59
		Nucleus caudatus	59
		Putamen	59

^1^ The number of samples form control subjects included in the meta-analysis.

**Table 2:** Mouse transcriptomic GEO datasets meeting the inclusion criteria and included in the analysis.

Accession Number	Title	Samples	*n* ^1^
GSE107183	RNA changes in hippocampus of transgenic murine model of tauopathy (rTg4510 mice) compared to controls at asymptomatic stage (2 months) of neurodegeneration as determined by mRNA deep sequencing.	Hippocampus	6
GSE112575	Frontal cortex transcriptomic analysis of a TDP-43 Q331K knock-in mouse (20 month)	Frontal cortex	8
GSE116752	Striatal transcriptome of a mouse model of ADHD reveals a pattern of synaptic remodeling	Hippocampus	5
GSE126814	Quantitative Analysis of Wild Type and Neat1 -/- Cerebral Frontal Cortex Transcriptomes	Frontal cortex	5
GSE130254	Regional Analysis of The Brain Transcriptome in Mice Bred For High And Low Methamphetamine Consumption	Prefrontal cortex	48
		Nucleus accumbens	47
		Ventral midbrain	48
GSE136869	Transcriptome analysis using RNA sequencing of the hippocampus of aged LPAR2-/- versus wildtype control mice	Hippocampus	7
GSE146628	Identification of Natural Antisense Transcripts in Mouse Brain and Their Association with Autism Spectrum Disorder Risk Genes	Prefrontal cortex	12
		Striatum	12
GSE147842	Adult mouse hippocampal transcriptome changes associated with long-term behavioral and metabolic effects of gestational air pollution toxicity	Hippocampus	10
GSE148075	Wild mice with different social network sizes vary in brain gene expression	Prefrontal cortex	29
		Hippocampus	29
		Hypothalamus	28
GSE166831	Altered hippocampal transcriptome dynamics following sleep deprivation	Hippocampus	9
GSE170997	Transcriptomics data of blood and brain from the YAC128 Huntington’s disease mouse model (brain)	Striatum	8
		Cerebellum	8
GSE99353	Frontal cortex transcriptomic analysis of a TDP-43 Q331K knock-in mouse (5 month)	Prefrontal cortex	6

^1^ The number of control wild type samples without mutations, knock-ins, etc., included in the meta-analysis.

**Table 3:** Datasets for the specific cellular or subcellular fractions included in the review.

Accession Number	Title	Samples	*n* ^1^
GSE110727	Characterization of the nuclear and cytosolic transcriptomes in human brain tissue	Frontal cortex (human)	12
GSE121199	Biology and Bias in Cell Type-Specific RNAseq of Nucleus Accumbens Medium Spiny Neurons	Nucleus accumbens (mouse)	9
GSE130376	Next-generation sequencing of cholinergic interneurons in the nucleus accumbens of cocaine-addicted and non-addicted mice	Nucleus accumbens (mouse)	49

^1^ The number of samples included in the meta-analysis.

## Data Availability

The data are available in the GEO database (https://www.ncbi.nlm.nih.gov/geo/ (accessed on 23 March 2021, the detailed information is listed in Tables S1–S3). The Genotype-Tissue Expression (GTEx) data from dbGaP accession number phs000424.v8.p2 are available at the GTEx Portal (https://www.gtexportal.org/home/, (accessed on 22 March 2022). The ISH data are available at the Allen Mouse Brain Atlas (https://mouse.brain-map.org accessed on 16 March 2022), including experiment 70724989, riboprobe RP_050705_03_A11, sagittal projection (available at https://mouse.brain-map.org/experiment/show/70724989, accessed on 16 March 2022); experiment 68744522, riboprobe RP_050208_01_A11, sagittal projection (available at https://mouse.brain-map.org/experiment/show/68744522 accessed on 16 March 2022); experiment 358, riboprobe RP_Baylor_102735, sagittal projection (available at https://mouse.brain-map.org/experiment/show/358 accessed on 16 March 2022); and experiment 79394355, riboprobe RP_071018_03_D06, sagittal projection (available at https://mouse.brain-map.org/experiment/show/79394355 accessed on 16 March 2022).

## Supplementary Materials

The following supporting information can be downloaded at: https://www.mdpi.com/article/10.3390/biom12091259/s1, Table S1: Human transcriptomic GEO datasets meeting the inclusion criteria and included in the analysis, Table S2: Mouse transcriptomic GEO datasets meeting the inclusion criteria and included in the analysis, Table S3: Datasets for the specific cellular or subcellular fractions included in the review, Table S4: Genes co-expressed with TAAR6 in MSN-D1, MSN-D2, and cholinergic interneurons, Figure S1: Full-length agarose gel: reverse transcription-polymerase chain reaction (RT-PCR) with mouse TAAR6 and GAPDH (housekeeping gene) specific primers using RNA isolated from the prefrontal cortex and olfactory bulb of 6 male animals confirmed TAAR6 mRNA expression.
