# Supplementary material for: Public Transcriptomic Data Meta-Analysis Demonstrates TAAR6 Expression in the Mental Disorder-Related Brain Areas in Human and Mouse Brain
Source: Biomolecules. 2022 Sep 7;12(9):1259. doi: 10.3390/biom12091259 (PMC9496192; doi:10.3390/biom12091259)
Supplement: Supplementary file 1 [file biomolecules-12-01259-s001.zip › biomolecules-1837560-supplementary.pdf]

*Article*

# Public Transcriptomic Data Meta-Analysis Demonstrates TAAR6 Expression in the Mental Disorder-Related Brain Areas in Human and Mouse Brain

**Anastasia N. Vaganova [1], Nataliia V. Katolikova [1], Ramilya Z. Murtazina [1], Savelii R. Kuvarzin [1] and Raul R. Gainetdinov [1,2,*]**

[1] Institute of Translational Biomedicine, St. Petersburg State University, Universitetskaya Nab. 7/9, 199034 St. Petersburg, Russia
[2] St. Petersburg University Hospital, St. Petersburg State University, Universitetskaya Nab. 7/9, 199034 St. Petersburg, Russia
* Correspondence: gainetdinov.raul@gmail.com

**Table S1.** Human transcriptomic GEO datasets meeting the inclusion criteria and included in the analysis.

| Accession Number | Title | Samples | Link |
|---|---|---|---|
| GSE53239 | RNA-sequencing of the brain transcriptome implicates dysregulation of neuroplasticity, circadian rhythms, and GTPase binding in bipolar disorder | Dorsolateral prefrontal cortex | https://www.ncbi.nlm.nih.gov/geo/query/acc.cgi?acc=GSE53239 |
| GSE53697 | RNAseq in Alzheimer's Disease patients | Dorsolateral prefrontal cortex | https://www.ncbi.nlm.nih.gov/geo/query/acc.cgi?acc=GSE53697 |
| GSE110727 | Characterization of the nuclear and cytosolic transcriptomes in human brain tissue | Frontal cortex | https://www.ncbi.nlm.nih.gov/geo/query/acc.cgi?acc=GSE110727 |
| GSE123496 | Human brain tissues from healthy controls and multiple sclerosis patients | Parietal cortex<br>Frontal cortex<br>Corpus callosum<br>Internal capsule<br>Hippocampus | https://www.ncbi.nlm.nih.gov/geo/query/acc.cgi?acc=GSE123496 |
| GSE124439 | Postmortem Cortex Samples Identify Distinct Molecular Subtypes of ALS: Retrotransposon Activation, Oxidative Stress, and Activated Glia | Frontal cortex<br>Motor cortex | https://www.ncbi.nlm.nih.gov/geo/query/acc.cgi?acc=GSE124439 |
| GSE160521 | Diurnal rhythms across the human dorsal and ventral striatum | Nucleus accumbens<br>Nucleus caudatus<br>Putamen | https://www.ncbi.nlm.nih.gov/geo/query/acc.cgi?acc=GSE160521 |

**Table S2.** Mouse transcriptomic GEO datasets meeting the inclusion criteria and included in the analysis.

| Accession Number | Title | Samples | Study Groups |
|---|---|---|---|
| GSE107183 | RNA changes in hippocampus of transgenic murine model of tauopathy (rTg4510 mice) compared to controls at asymptomatic stage (2 months) of neurodegeneration as determined by mRNA deep sequencing. | Hippocampus | https://www.ncbi.nlm.nih.gov/geo/query/acc.cgi?acc=GSE107183 |
| GSE112575 | Frontal cortex transcriptomic analysis of a TDP-43 Q331K knock-in mouse [20month] | Frontal cortex | https://www.ncbi.nlm.nih.gov/geo/query/acc.cgi?acc=GSE112575 |
| GSE116752 | Striatal transcriptome of a mouse model of ADHD reveals a pattern of synaptic remodeling | Striatum | https://www.ncbi.nlm.nih.gov/geo/query/acc.cgi?acc=GSE116752 |
| GSE126814 | Quantitative Analysis of Wild Type and Neat1 -/- Cerebral Frontal Cortex Transcriptomes | Frontal cortex | https://www.ncbi.nlm.nih.gov/geo/query/acc.cgi?acc=GSE126814 |
| GSE130254 | Regional Analysis of The Brain Transcriptome In Mice Bred For High And Low Methamphetamine Consumption | Prefrontal cortex<br>Nucleus accumbens<br>Ventral Midbrain | https://www.ncbi.nlm.nih.gov/geo/query/acc.cgi?acc=GSE130254 |

| | | | |
|---|---|---|---|
| GSE136869 | Transcriptome analysis using RNA sequencing of the hippocampus of aged LPAR2-/- versus wildtype control mice | Hippocampus | https://www.ncbi.nlm.nih.gov/geo/query/acc.cgi?acc=GSE136869 |
| GSE146628 | Identification of Natural Antisense Transcripts in Mouse Brain and Their Association with Autism Spectrum Disorder Risk Genes | Prefrontal cortex<br>Striatum | https://www.ncbi.nlm.nih.gov/geo/query/acc.cgi?acc=GSE146628 |
| GSE147842 | Adult mouse hippocampal transcriptome changes associated with long-term behavioral and metabolic effects of gestational air pollution toxicity | Hippocampus | https://www.ncbi.nlm.nih.gov/geo/query/acc.cgi?acc=GSE147842 |
| GSE148075 | Wild mice with different social network sizes vary in brain gene expression | Prefrontal cortex<br>Hippocampus<br>Hypothalamus | https://www.ncbi.nlm.nih.gov/geo/query/acc.cgi?acc=GSE148075 |
| GSE166831 | Altered hippocampal transcriptome dynamics following sleep deprivation | Hippocampus | https://www.ncbi.nlm.nih.gov/geo/query/acc.cgi?acc=GSE166831 |
| GSE170997 | Transcriptomics data of blood and brain from the YAC128 Huntington's disease mouse model [brain] | Cerebellum<br>Striatum | https://www.ncbi.nlm.nih.gov/geo/query/acc.cgi?acc=GSE170997 |
| GSE99353 | Frontal cortex transcriptomic analysis of a TDP-43 Q331K knock-in mouse [5month] | Frontal cortex | https://www.ncbi.nlm.nih.gov/geo/query/acc.cgi?acc=GSE99353 |

**Table S3.** Datasets for the specific cellular or subcellular fractions included in the review.

| Accession Number | Title | Samples | Link |
|---|---|---|---|
| GSE110727 | Characterization of the nuclear and cytosolic transcriptomes in human brain tissue | Frontal cortex (human) | https://www.ncbi.nlm.nih.gov/geo/query/acc.cgi?acc=GSE5110727 |
| GSE121199 | Biology and Bias in Cell Type-Specific RNAseq of Nucleus Accumbens Medium Spiny Neurons | Nucleus accumbens (mouse) | https://www.ncbi.nlm.nih.gov/geo/query/acc.cgi?acc=GSE121199 |
| GSE130376 | Next-generation sequencing of cholinergic interneurons in the nucleus accumbens of cocaine-addicted and non-addicted mice | Nucleus accumbens (mouse) | https://www.ncbi.nlm.nih.gov/geo/query/acc.cgi?acc=GSE130376 |

**Figure S1.** Full-length agarose gel: reverse transcription-polymerase chain reaction (RT-PCR) with mouse TAAR6 and GAPDH (housekeeping gene) specific primers using RNA isolated from the prefrontal cortex (lanes 1-6 for TAAR6, 7-12 for GAPDH) and olfactory bulb (lanes 15-20 for TAAR6, and 21-26 for GAPDH) of 6 male animals confirmed TAAR6 mRNA expression. Amplicons from olfactory epithelium cDNA (lane 13) and plasmid containing mouse Taar6 gene sequence (lane 14) were included as primer specificity controls. Negative control is K-, DNA ladder is M (Evrogen, #NL003). Red is a source of cropped image. Gel Doc XR+ Documentation System (Bio-Rad) was used for agarose EtBr-stained gel analysis. Time of imaging exposure was 0.936 sec.

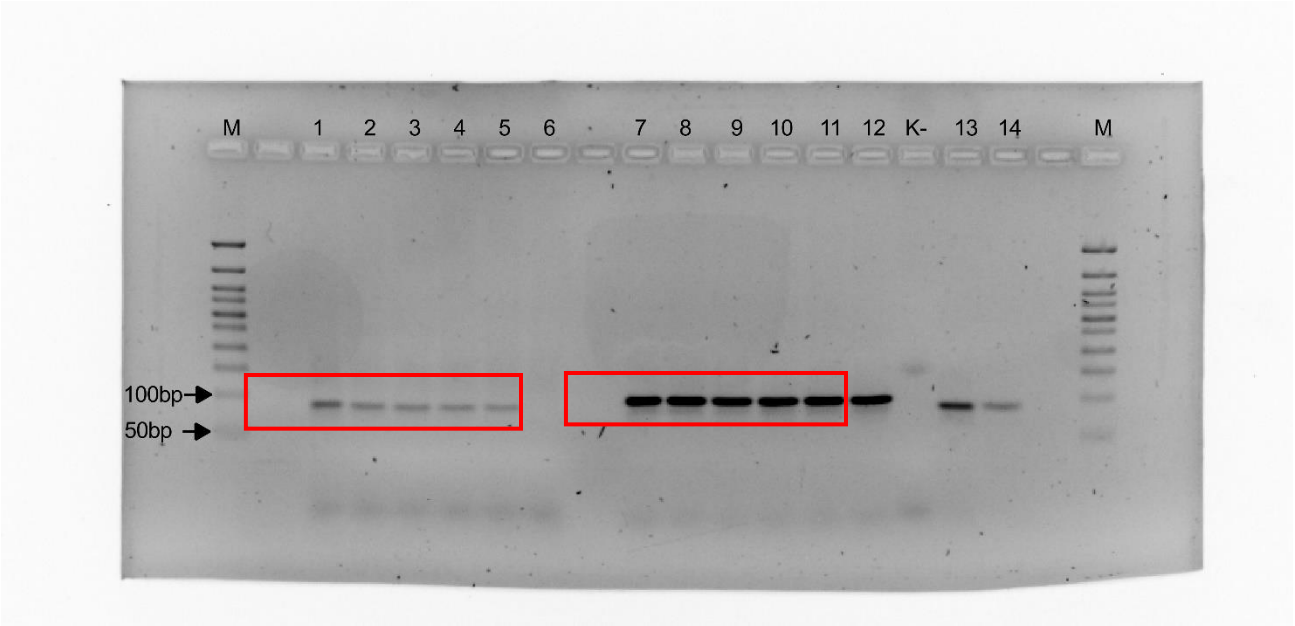

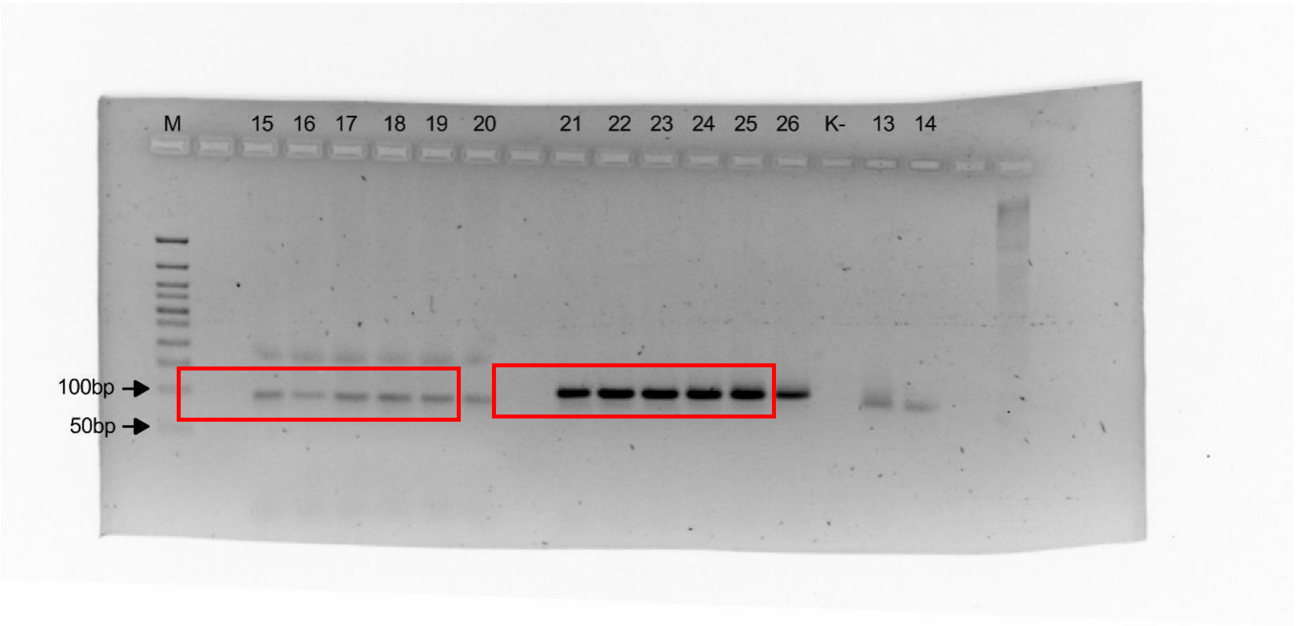
M
15
16
17
18
19
20
21
22
23
24
25
26
K-
13
14
100bp
50bp
